# EFFECT OF COMBINED EXPOSURE TO LEAD, MERCURY, AND CADMIUM ON HYPERTENSION: THE 2008–2013 KOREAN NATIONAL HEALTH AND NUTRITION EXAMINATION SURVEYS

**DOI:** 10.13075/ijomeh.1896.02524

**Published:** 2025

**Authors:** Doh Hee Kim, Seunghee Lee, Mijung Jang, Kyoosang Kim

**Affiliations:** 1 Seoul Medical Center, Department of Research Institute, Seoul, Republic of Korea; 2 Seoul Medical Center, Department of Occupational Environmental Medicine, Seoul, Republic of Korea

**Keywords:** hypertension, cadmium, mercury, lead, heavy metal exposure, Korean National Health and Nutrition Examination surveys

## Abstract

**Objectives:**

Many studies have demonstrated the effects of heavy metals on hypertension. However, to date, no study has used the Korean National Health and Nutrition Examination Survey (KNHANES) to analyze the effects of combined exposure to heavy metals on hypertension. Therefore, this study inferred the study population using weights based on a rolling sample and used standardized scores to estimate the effects of combined exposure to heavy metals on hypertension.

**Material and Methods:**

The authors used raw participant data from KNHANES (2008–2013), when heavy metal levels in the blood were measured. The authors analyzed the effects of lead, mercury, and cadmium on hypertension. The authors calculated standardized scores based on a regression analysis to analyze the effects of combined exposure. The odds ratios (ORs) of hypertension due to heavy metals were calculated using multivariate logistic regression, with the lowest quartile as the reference category.

**Results:**

In the crude regression analysis, lead, mercury, and cadmium exposure were associated with significant differences in the rate of hypertension (p < 0.05). However, when other heavy metals were included as covariates, only lead (OR = 1.29, 95% confidence interval [CI]: 1.08–1.55) and cadmium (OR = 1.47, 95% CI: 1.24–1.74) showed significant effects (p < 0.01). When the authors analyzed the effects of combined exposure to heavy metals, the OR of hypertension for the highest quartile, relative to the lowest quartile, was 1.78 (95% CI: 1.50–2.11, p < 0.001). Linear regression analysis revealed that heavy metal exposure was significantly associated with hypertension prevalence (p < 0.001).

**Conclusions:**

This study verified that heavy metal exposure affects the prevalence of hypertension and that combined exposure to multiple heavy metals is associated with a higher risk of hypertension than exposure to a single heavy metal. Further research is necessary to screen for heavy metal-related risk factors and evaluate whether the interactions between heavy metals are positive or negative.

## Highlights

- A novel national-scale analysis of heavy metals and hypertension risk were used.
- Lead (Pb) and cadmium (Cd) showed the strongest association with hypertension.
- Mercury had a weaker link to hypertension compared to Pb and Cd.
- Combined exposure to heavy metals revealed significant hypertension risk.
- Findings emphasize the need for monitoring heavy metal exposure for cardiovascular disease prevention.

## INTRODUCTION

Cardiovascular disease (CVD) affects approx. 523 million patients worldwide, with 18.6 million deaths attributed to CVD-related diseases annually [1]. Hypertension is a major cause of CVD and is closely related not only to heart failure, stroke, and chronic kidney disease [2]. When evaluating the potential health risks for CVD, it is essential to carefully consider factors such as social and personal activities and heavy metal exposure. Despite advances in healthcare technology and medical science, there are still unexplained risk factors for diseases, and exposure to heavy metals is known to be a major risk factor [3]. In the last 20 years, the rapid increase in research on CVD risk due to environmental exposure to heavy metals has provided evidence of the increasing risk of exposure to heavy metals due to industrialization [4].

The 1974 Food and Agriculture Organization of the United Nations (FAO)/World Health Organization (WHO) Joint Expert Committee on Food Additives (JECFA) included heavy metals, such as cadmium (Cd), lead (Pb), mercury (Hg), and arsenic (As), as important chemical contaminants to be monitored [5]. The Ministry of Environment of South Korea also selected Cd, Pb, and Hg as priority targets for the assessment of health risks. In particular, Pb, Hg, and Cd are known for their environmental contamination, long-term effects on the body, and cardiovascular toxicity. Even at low concentrations, Pb increases blood pressure and the risk of CVD [6]. Mercury is directly related to CVD and has also been reported to be closely related to the inflammatory response, which in turn is involved in CVD [7]. Cadmium acts as a catalyst for reactive oxygen species (ROS) production and, like Pb, increases oxidative stress by increasing ROS levels via the inhibition of glutathione and protein binding. This increases lipid peroxidation and reduces the phosphorylation of endothelial nitric oxide synthase, thereby affecting vascular diseases [8].

The Korea National Health and Nutrition Examination Survey (KNHANES) is a comprehensive study that includes a self-report health questionnaire, health examination, and nutritional intake and heavy metals survey in a sample designed to be representative of the whole nation. To date, while studies have used the KNHANES to investigate heavy metals and health, to the best of the authors’ knowledge, no studies have reported inferences at a national scale to investigate the effects of combined exposure to heavy metals on hypertension.

Therefore, this study aimed to perform weighted inference from the KNHANES data, assess the risk of hypertension, a major risk factor for CVD associated with individual heavy metals, and estimate the effect of combined exposure to heavy metals on hypertension risk. The authors’ findings provide valuable insights into the potential health risks of heavy metal exposure, particularly in relation to hypertension, a significant risk factor for cardiovascular disease.

## MATERIAL AND METHODS

### Materials

For the present study, the authors obtained raw data from KNHANES, a national-scale survey, with official permission. The authors only used data from 2008–2013, the period when heavy metal levels and blood pressure were measured. A total of 53 829 people participated in this study. After excluding 13 501 people <19 years old, 40 328 were included in the analysis. Sociodemographic factors and health behaviors were assessed using a self-report questionnaire. This careful selection process ensured the robustness of the findings.

### Blood pressure measurement

Blood pressure was measured using a Baumanometer (Baumanometer Desk model 0320 in 2007–2012 and Baumanometer Wall Unit 33(0850) in 2013–2015; W.A. Baum Co. Inc., New York, USA). Three measurements were obtained from the right arm of each participant. Hypertension was defined as a systolic blood pressure ≥140 mm Hg or a diastolic blood pressure ≥90 mm Hg, a self-report-ed diagnosis of hypertension by a physician, or the use of antihypertensive medication. The diagnosis of hypertension was based on the American Heart Association criteria from 2003–2016 [9].

### Measurement of heavy metal levels

For the heavy metal analysis, 5 ml of whole blood was collected in a trace-element EDTA tube. Pretreatment for the heavy metal analysis was performed by adding whole blood to the matrix modifier reagent (for Pb, Triton X-100 and ammonium hydrogen phosphate dibasic; for Cd, Triton X-100, ammonium hydrogen phosphate dibasic, magnesium nitrate, and ammonium hydrogen phosphate monobasic). Mercury was analyzed by direct injection into the instrument without pretreatment. To measure the heavy metal levels in the blood, Pb and Cd were analyzed using graphite furnace atomic absorption spectrometry (GFAAS) (Perkin Elmer AAnalyst 600, Perkin Elmer, Turku, Finland) using the Zeeman method of background correction. Mercury was analyzed using the gold amalgam method with DMA-80 (Milestone, Sorisole, Italy). The basic operational parameters were optimized for Pb (wavelength: 283 nm, slit width: 0.7 nm, lamp current: 440 mA), Cd (wavelength: 228.8 nm EDL lamp, slit width: 0.7 nm, lamp current: 220 mA), and Hg (wavelength: 253.7 nm). For internal quality assurance and control, commercial standard substances (Lyphochek Whole Blood Metals Control, lot No. 36701, 36711, 36721, 36731, 36741, and 36751, Bio-Rad, Hercules, CA, USA) were used. The limit of detection was 0.207 µg/dl for Pb, 0.158 µg/l for Hg, and 0.081 µg/l for Cd. All samples exceeded these limits of detection. The inter-assay coefficients of variation were 2.65–6.50% for Pb, 0.47–6.08% for Hg, and 0.95–4.82% for Cd. All blood heavy metal analyses were conducted by the Neodin Medical Institute following an external quality assessment scheme in Germany (Friedrich-Alexander University) and a quality assurance program in South Korea (Korea Occupational Safety and Health Agency).

### Complex sample analysis and weights

The KNHANES survey participants were selected annually through multistage stratified cluster random sampling; therefore, the authors implemented an analysis plan and applied weights to the years 2008–2013 in accordance with the methods suggested by the KNHANES [10]. When generating an analysis using only a portion of the data, the omission of some complex sampling data can lead to bias in the standard error of the estimates. Therefore, in this study, the authors generated grouping variables designated as populations and performed complex sampling with the grouping variables included as stratification variables when generating the analysis plan.

### Covariates

The sociodemographic factors analyzed were sex, age, region, occupation, and education, and the health behaviors analyzed were smoking, alcohol consumption, physical activity, and obesity. The heavy metals were Pb, Hg, and Cd, and hypertension was the target disease. Age was categorized as 20–39 years, 40–59 years, or ≥60 years. The analysis was restricted to adults (≥20 years). Regions were categorized into major cities (urban: Seoul, Busan, Daegu, Incheon, Gwangju, and Daejeon Ulsan) and provinces (countryside: Gyeonggi-do, Gangwon-do, Chungcheongbuk-do, Chungcheongnam-do, Jeollabuk-do, Jeollanam-do, Gyeongsangbuk-do, Gueongsangnam-do, and Jeju-do). Occupation was categorized into white-collar jobs (professional, clerical, and service), blue-collar jobs (agriculture, forestry and fishery, crafts and trade, simple labor), and others (homemakers and students). Education was categorized as middle school graduate or below, high school graduate, or university graduate or above. Income was divided into low, middle-low, middle-high, and high quartiles. Smoking was categorized as non-smoking if not applicable, smoking for persons who reported smoking or sometimes smoking, and past smoking for persons who had previously smoked but did not currently smoke. Alcohol consumption was categorized as “yes” or “no” according to lifetime drinking experience. Physical activity, based on walking activity, was categorized as “yes” or “no,” depending on whether the participant walked for at least 150 min/week. Across all data, responses of “not sure” or “no response” were treated as missing values.

### Heavy metal ranges

The authors estimated the range of heavy metal distributions across the entire population. For Pb and Cd, the authors set the ranges using all measured data and omitted missing values. However, for Hg, the maximum value (168.49 µg/l) was very high compared with the other observed values (0.336–60.678 µg/l); therefore, the authors treated this as an anomaly and omitted it from the analysis. The authors divided the entire adult population into quartiles based on heavy metal exposure.

### Heavy metal standardized scores

To estimate the effects of combined exposure to heavy metals, the authors generated standardized heavy metal scores using Z-scores, which were calculated by subtracting the mean (M) and dividing it by the standard deviation (SD). To assess the risk associated with the combined exposure to heavy metals, the authors first developed a combined exposure score by adding the standardized Z-scores of each heavy metal. The authors then calculated quartiles to convert the combined exposure score into a discrete variable, which was used in the regression analysis. Owing to the standardization process, the Z-scores had M = 0 and SD = 1.

### Ethics approval

The KNHANES was conducted in accordance with the Declaration of Helsinki and approved by the Institutional Review Board of the Korea Center for Disease Control (IRB No. 1401-047-547) and Seoul Medical Center (IRB No. 2024-07-003). Informed consent was obtained from all participants involved in the KNHANES.

### Statistical analysis

To compare heavy metal levels according to sociodemographic factors, health behaviors, and hypertension, the authors determined the geometric M using the F-test. The authors performed 3 types of logistic regression analysis to analyze the effects of heavy metals on hypertension. First, the authors analyzed the risk associated with exposure to a single heavy metal without including other heavy metals as covariates. Second, the authors analyzed the risk associated with each heavy metal, including other heavy metals, as covariates. Finally, the authors analyzed the risk of hypertension using the combined exposure score based on standardized scores for each heavy metal as the independent variable. In the regression analysis, using the lowest quartile (Q1) as the reference level, the odds ratios (ORs) for hypertension associated with each heavy metal, 95% confidence intervals (CI), and p values were calculated. Three types of logistic regression models were used. Model 1 was a crude model using only the heavy metals as the independent variables. Model 2 included sociodemographic factors as covariates, and model 3 included both sociodemographic factors and health behaviors as covariates to analyze the effects of heavy metals on hypertension. In the logistic regression analysis, the authors tested the significance of linear trends in the ORs for hypertension prevalence associated with the different quartiles of heavy metal exposure. In accordance with the complex sample analysis guidelines in the KNHANES, “system missing values,” “not applicable,” and “not sure” were treated as missing values. The authors included these to prevent bias in the standard error of estimates due to missing complex sample design data. IBM SPSS 23K (Armonk, NY, USA) was used for the statistical analysis.

## RESULTS

### General characteristics of the participants

Table 1 describes the general characteristics of the KNHANES in the 6 years from 2008 to 2013. The authors also show the inferred values for the entire Korean population after applying the household survey weights to the measured values. The sex ratio, age, region, and education of the inferred population did not show significant differences by category. However, the proportion of current or past smokers was approx. 44%, and the proportion of participants with alcohol consumption was approx. 87%. Approximately 32% of the participants were obese, and approx. 9% practiced physical activity based on walking. The prevalence of hypertension among the inferred population was high (approx. 50%).

**Table 1.** General characteristics of study participants in Korean National Health and Nutrition Examination Survey, 2008–2013

Variable	Participants (N = 40 328, WN = 38 232 401)	
	n (wn)	% (w%)*
Sociodemographic		
sex		
male	17 412 (18 923 184)	43.2 (49.50)
female	22 916 (19 309 217)	56.8 (50.50)
age		
20–39 years	12 504 (14 977 611)	31.01 (39.17)
40–59 years	15 028 (15 417 894)	37.26 (40.33)
≥60 years	12 796 (7 836 896)	31.73 (20.50)
region		
urban	18 243 (18 268 266)	45.24 (47.78)
countryside	22 085 (19 964 135)	54.76 (52.22)
occupation		
white-collar job	11 976 (13 915 072)	29.70 (36.40)
blue-collar job	9 804 (9 956 757)	24.31 (26.04)
other job	15 198 (13 261 011)	37.69 (34.69)
education		
middle school	14 056 (11 033 995)	34.85 (28.86)
high school	12 343 (14 417 002)	30.61 (37.71)
university	10 670 (11 811 994)	26.46 (30.90)
income		
low	9 828 (10 042 874)	24.37 (26.27)
middle low	9 934 (9 515 068)	24.63 (24.89)
middle high	9 945 (9 343 909)	24.66 (24.44)
high	9 937 (8 896 360)	24.64 (23.27)
Health behaviors		
smoking		
smoking	10 596 (11 571 261)	26.27 (30.27)
past smoking	4 632 (5 307 946)	11.49 (13.88)
no smoking	21 874 (20 456 143)	54.24 (53.50)
alcohol consumption		
no	5 310 (4 112 354)	13.17 (10.76)
yes	31 779 (33 221 071)	78.80 (86.89)
obesity		
no	25 792 (25 856 376)	63.96 (67.63)
yes	12 101 (12 239 486)	30.01 (32.01)
physical activity		
no	27 724 (33 648 868)	68.75 (88.01)
yes	3 458 (3 589 906)	8.57 (9.39)
hypertension		
no	16 295 (19 132 598)	40.41 (50.04)
yes	20 730 (19 023 985)	51.40 (49.76)

wn – weighted sample size; w% – weighted percentage.

White-collar jobs – professional, office, and service jobs; blue-collar jobs – agriculture, laborers, and simple labor; other jobs – homemaker and student. Income groups: low – <0.75 million KRW; middle low – 0.75–1.49 million KRW; middle high – 1.50–2.46 million KRW; high – >2.46 million KRW.

* Percentages and weighted percentages were calculated including missing values (missing values was not shown in the table).

### Comparison of Pb, Hg, and Cd according to general characteristics

Table 2 provides the results of the F-test to analyze the differences in the levels of heavy metals in the blood according to general characteristics. As the general characteristics included temporal factors, the authors compensated for this by using the geometric M in the analysis. Blood Pb levels showed significant differences according to general characteristics (p < 0.0001). Mercury showed significant differences according to most general characteristics (p < 0.0001) but did not show any significant differences by region or physical activity. Cadmium was similar to Hg, showing significant differences for most general characteristics (p < 0.001) but not for obesity or physical activity. Thus, Pb, Hg, and Cd showed significant differences according to sex, age, occupation, education, income, smoking, alcohol consumption, and hypertension (p < 0.0001).

**Table 2.** Comparison of heavy metal levels by participant characteristics in Korean National Health and Nutrition Examination Survey, 2008–2013

Variable	Lead [μg/dl]		p	Mercury [μg/l]		p	Cadmium [μg/l]		p
	GM	GSD		GM	GSD		GM	GSD	
Sociodemographic									
sex									
male	2.5	1.007	<0.0001***	4.42	1.012	<0.0001***	0.88	1.01	<0.0001***
female	1.84	1.007		3.14	1.011		1.04	1.01	
age									
20–39 years	1.84	1.008	<0.0001***	3.37	1.011	<0.0001***	0.71	1.012	<0.0001***
40–59 years	2.36	1.007		4.23	1.012		1.13	1.009	
≥60 years	2.38	1.012		3.49	1.022		1.23	1.014	
region									
urban	2.09	1.008	<0.0001***	3.72	1.013	0.9373	0.93	1.011	0.0008***
countryside	2.2	1.008		3.72	1.014		0.98	1.011	
occupation									
white-collar job	2.05	1.008	<0.0001***	4.03	1.012	<0.0001***	0.87	1.011	<0.0001***
blue-collar job	2.52	1.01		3.99	1.018		1.07	1.013	
other job	1.98	1.009		3.24	1.014		0.99	1.013	
education									
middle school	2.43	1.01	<0.0001***	3.6	1.018	0.0002***	1.26	1.011	<0.0001***
high school	2.11	1.008		3.67	1.012		0.92	1.012	
university	1.94	1.009		3.89	1.013		0.78	1.012	
income									
low	2.22	1.01	<0.0001***	3.44	1.017	<0.0001***	1.02	1.014	<0.0001***
middle low	2.17	1.011		3.62	1.015		0.98	1.014	
middle high	2.09	1.01		3.71	1.016		0.94	1.014	
high	2.09	1.011		4.2	1.016		0.9	1.016	
Health behaviors									
smoking									
past smoking	2.33	1.013	<0.0001***	4.08	1.021	<0.0001***	0.82	1.016	<0.0001***
no smoking	1.89	1.007		3.27	1.011		0.93	1.01	
smoking	2.57	1.008		4.49	1.014		1.09	1.012	
alcohol consumption									
no	1.99	1.016	<0.0001***	3.32	1.028	<0.0001***	1.16	1.02	<0.0001***
yes	2.16	1.006		3.77	1.009		0.94	1.008	
obesity									
no	2.1	1.007	<0.0001***	3.5	1.01	<0.0001***	0.95	1.009	0.0611
yes	2.24	1.009		4.22	1.014		0.98	1.014	
physical activity									
no	2.12	1.006	<0.0001***	3.71	1.01	0.1443	0.96	1.008	0.098
yes	2.33	1.019		3.84	1.025		1	1.023	
Disease									
hypertension									
no	1.96	1.008	<0.0001***	3.43	1.011	<.0001***	0.87	1.01	<.0001***
yes	2.35	1.007		4.04	1.013		1.06	1.01	

GM – geometric mean; GSD – geometric standard deviation; WN – weighted sample size.

p-values were calculated for differences in the geometric means (GM) based on categorical variables using the F-test.

*** p < 0.001.

### Effects of single heavy metals on hypertension

First, the authors analyzed the effects of single heavy metals without including other heavy metals as covariates. This analysis used 3 models with different independent variables:

– heavy metals,
– heavy metals + sociodemographic factors,
– heavy metals + sociodemographic factors + health behaviors (Table 3).

**Table 3.** Effects of single heavy metals, heavy metals with other heavy metals as covariates, and combined exposure to heavy metals on hypertension participants in Korean National Health and Nutrition Examination Survey, 2008–2013

Variable	Model 1		Model 2		Model 3	
	OR (95% CI)	p (trend^a^)	OR_a_ (95% CI)	p (trend^a^)	OR_a_ (95% CI)	p (trend^a^)
Single heavy metal						
lead						
Q1 (<1.62 μg/dl)	1 (ref.)	<0.0001 (<0.0001)***	1 (ref.)	<0.0001 (<0.0001)***	1 (ref.)	<0.0001 (<0.0001)***
Q2 (>1.62–2.16 μg/dl)	1.55 (1.340–1.782)		1.16 (1.005–1.344)		1.14 (0.985–1.325)	
Q3 (>2.16–2.84 μg/dl)	2.24 (1.954–2.567)		1.38 (1.183–1.597)		1.38 (1.181–1.603)	
Q4 (>2.84 μg/dl)	3.15 (2.737–3.619)		1.40 (1.184–1.659)		1.45 (1.219–1.730)	
mercury						
Q1 (<2.34 μg/l)	1 (ref.)	<0.0001 (<0.0001)***	1 (ref.)	<0.0001 (<0.0001)***	1 (ref.)	0.0043 (0.0004)**
Q2 (>2.34–3.61 μg/l)	1.09 (0.950–1.250)		1.07 (0.914–1.245)		1.05 (0.897–1.224)	
Q3 (>3.61–5.52 μg/l)	1.47 (1.282–1.674)		1.31 (1.129–1.516)		1.22 (1.052–1.415)	
Q4 (>5.52 μg/l)	1.93 (1.685–2.204)		1.42 (1.214–1.659)		1.29 (1.102–1.514)	
cadmium						
Q1 (<0.67 μg/l)	1 (ref.)	<0.0001 (<0.0001)***	1 (ref.)	<0.0001 (<0.0001)***	1 (ref.)	<0.0001 (<0.0001)***
Q2 (>0.67–1.44 μg/l)	1.47 (1.300–1.666)		1.20 (1.041–1.380)		1.26 (1.087–1.450)	
Q3 (>1.00–1.44 μg/l)	1.93 (1.701–2.178)		1.34 (1.160–1.557)		1.43 (1.224–1.661)	
Q4 (>1.44 μg/l)	2.24 (1.957–2.558)		1.46 (1.250–1.714)		1.61 (1.365–1.895)	
Heavy metal with other heavy metals as covariates						
lead						
Q1 (<1.62 μg/dl)	1 (ref.)	<0.0001 (<0.0001)***	1 (ref.)	0.0284 (0.0073)*	1 (ref.)	0.0099 (0.0016)**
Q2 (>1.62–2.16 μg/dl)	1.39 (1.204–1.608)		1.10 (0.947–1.275)		1.08 (0.929–1.259)	
Q3 (>2.16–2.84 μg/dl)	1.88 (1.626–2.164)		1.25 (1.071–1.461)		1.26 (1.077–1.476)	
Q4 (>2.84 μg/dl)	2.46 (2.118–2.860)		1.23 (1.033–1.47)		1.29 (1.075–1.548)	
mercury						
Q1 (<2.34 μg/l)	1 (ref.)	<0.0001 (<0.0001)***	1 (ref.)	0.0016 (0.0001)**	1 (ref.)	0.1079 (0.0196)
Q2 (>2.34–3.61 μg/l)	0.98 (0.850–1.132)		1.03 (0.881–1.206)		1.00 (0.857–1.176)	
Q3 (>3.61–5.52 μg/l)	1.21 (1.051–1.391)		1.23 (1.061–1.434)		1.14 (0.980–1.326)	
Q4 (>5.52 μg/l)	1.42 (1.228–1.638)		1.31 (1.113–1.534)		1.17 (0.995–1.381)	
cadmium						
Q1 (<0.67 μg/l)	1 (ref.)	<0.0001 (<0.0001)***	1 (ref.)	0.0055 (0.0006)**	1 (ref.)	<0.0001 (<0.0001)***
Q2 (>0.67–1.44 μg/l)	1.28 (1.121–1.452)		1.13 (0.983–1.308)		1.20 (1.037–1.390)	
Q3 (>1.00–1.44 μg/l)	1.55 (1.365–1.766)		1.24 (1.070–1.440)		1.34 (1.145–1.561)	
Q4 (>1.44 μg/l)	1.69 (1.459–1.946)		1.32 (1.118–1.548)		1.47 (1.238–1.737)	
Combined exposure to heavy metals						
Q1 (<0.34 μg/l)	1 (ref.)	<0.0001 (<0.0001)***	1 (ref.)	<0.0001 (<0.0001)***	1 (ref.)	<0.0001 (<0.0001)***
Q2 (>0.34–0.98 μg/l)	2.03 (1.770–2.334)		1.44 (1.233–1.674)		1.41 (1.212–1.647)	
Q3 (>0.98–3.36 μg/l)	2.69 (2.360–3.070)		1.61 (1.384–1.875)		1.61 (1.378–1.877)	
Q4 (>3.36 μg/l)	3.67 (3.208–4.202)		1.77 (1.499–2.081)		1.78 (1.502–2.111)	

OR_a_ – adjusted odds ratio for confounders

Confidence interval by trend; significant differences in the heavy metal exposure range by linear regression analysis.

Model 1 – the crude model; model 2 – adjusted for the sociodemographic factors of sex, age, region, occupation, education, and income; model 3 – further adjusted for the health behaviors of smoking, alcohol consumption, obesity, and physical activity.

* p < 0.05;

** p < 0.01;

*** p < 0.001.

a A trend test was performed by incorporating variables from the median of each quartile into the regression model.

The authors calculated ORs using the lowest quartiles for Pb, Hg, and Cd as reference levels and analyzed trends using linear regression analysis. All heavy metals showed significant differences in hypertension ORs and trends with increasing blood heavy metal levels (p < 0.0001). In model 1 of the effects of single heavy metals on hypertension, the OR of the highest quartile relative to the lowest quartile was the highest for Pb (OR = 3.15, 95% CI: 2.74–6.62, p < 0.0001), followed by Cd (OR = 2.24, 95% CI: 1.96–2.56, p < 0.0001), and Hg (OR = 1.93, 95% CI: 1.69–2.20, p < 0.0001). In model 2, accounting for sociodemographic factors, the highest OR was for Cd (OR = 1.46, 95% CI: 1.25–1.71, p < 0.0001), followed by Hg (OR = 1.42, 95% CI: 1.21–1.66, p < 0.0001), and Pb (OR = 1.40, 95% CI: 1.18–1.66, p < 0.0001). In model 3, accounting for all confounding factors, the highest OR was for Cd (OR = 1.61, 95% CI: 1.37–1.90, p < 0.0001), followed by Pb (OR = 1.45, 95% CI: 1.22–1.73, p < 0.0001), and Hg (OR = 1.29, 95% CI: 1.10–1.51, p = 0.0043).

### Effects of heavy metals on hypertension

The authors analyzed the hypertension ORs associated with different levels of heavy metal exposure using the model, with other heavy metals included as covariates (Table 3). Similar to the previous analysis, the authors used 3 models, varying according to the inclusion of sociodemographic factors and health behaviors, and analyzed the ORs for hypertension relative to the lowest quartiles. The hypertension ORs associated with heavy metals when other heavy metals were included as covariates were similar to the results in Table 3, which did not include other heavy metals as covariates. However, compared with the results in Table 3, the ORs for hypertension prevalence were lower. In model 1, relative to the lowest quartile (Q1), the OR of the highest quartile (Q4) was higher for Pb (OR = 2.46, 95% CI: 2.12–2.86, p < 0.0001) than for Cd (OR = 1.69, 95% CI: 1.46–1.95, p < 0.0001) or Hg (OR = 1.42, 95% CI: 1.23–1.64, p < 0.0001). In models 2 and 3, the ORs for Q4 were highest for Cd, at 1.32 (95% CI: 1.12–1.55, p < 0.0001) and 1.47 (95% CI: 1.24–1.74, p < 0.0001), respectively. For Pb, the OR of Q4 in model 2 decreased at 1.23 (95% CI: 1.03–1.47, p = 0.0284) but then increased again in model 3 to 1.29 (95% CI: 1.08–1.55, p = 0.0099). In contrast, Hg showed the lowest OR for Q4 in model 1, at 1.42 (95% CI: 1.23–1.64, p < 0.0001), and the OR decreased further with the inclusion of sociodemographic factors and health behaviors as covariates. In model 3, the OR for Hg-associated hypertension was not statistically significant.

### Effects of combined exposure to heavy metals on hypertension

The authors standardized exposure to individual heavy metals with respect to their properties, such as units, toxicity, and exposure time, by converting heavy metal levels to Z-scores using Ms and SDs, and then used the standardized scores to analyze the effects of combined exposure to heavy metals (Table 3). After calculating standardized scores for exposure to each of the 3 heavy metals (Pb, Hg, and Cd) in each participant, the authors added these scores, converted them to quartiles, and then analyzed the ORs relative to the lowest quartile (Q1). When the authors analyzed the effects of combined exposure to heavy metals on hypertension in the absence of other covariates, a higher combined level of heavy metals in the blood was associated with an increased OR, and the OR in Q4 was 3.67 (95% CI: 3.21–4.20, p < 0.0001) (model 1). When sociodemographic factors were included as covariates (model 2), the OR for hypertension prevalence associated with combined heavy metal exposure was 1.77 in Q4 (95% CI: 1.50–2.09). When the authors included both sociodemographic factors and health behaviors as covariates (model 3), the OR in Q4 was 1.78 (95% CI: 1.50–2.11, p < 0.0001). In all the regression models, increasing blood heavy metal levels were associated with significantly increasing trends in the prevalence of hypertension (trend <0.0001).

## DISCUSSION

Heavy metals are generally defined as metals with a density ≥5 g/cm^3^. This definition includes not only toxic metals, such as Pb, Hg, Cd, and As, but also essential trace metals, such as copper, magnesium, manganese, iron, and zinc. The FAO/WHO JEFCA presents provisional tolerable weekly intake (PTWI) values for human exposure to heavy metals of 25 μg/kg bw/week for Pb, 5.81 μg/kg bw/week for Cd, 5.0 μg/kg bw/week for Hg, and 1.6 μg/kg bw/week for methylmercury [11, 12]. However, owing to a lack of evidence for the Pb exposure threshold internationally, rescinding the PTWI and adopting a margin of exposure approach was recommended in 2010 [11]. Based on international limits, Hg has the highest toxicity in humans, followed by Cd and Pb. However, in the authors’ study, the comparison of blood concentrations of heavy metals and their effects on hypertension suggests that irrespective of general human toxicity or exposure, the risk can differ depending on the mechanisms by which heavy metals act on the body. Lead, Hg, and Cd are divalent toxic metals that are known to reduce the production and activity of antioxidant enzymes in cells through covalent bonding with sulfhydryl groups, leading to increased ROS levels. In addition, by increasing the expression of cellular adhesion molecules in vascular endothelial cells, heavy metals disrupt signal transmission and increase cell permeability, leading to increased oxidative stress and inflammation. This causes a pro-atherosclerotic response, promotes arteriosclerosis, and increases vascular contraction and local blood flow, thereby contributing to the onset of hypertension [13,14]. In the authors’ study, the OR for hypertension associated with combined exposure to heavy metals was higher than that for exposure to individual heavy metals, suggesting that interactions between different heavy metals can activate and exacerbate mechanisms that increase the risk of hypertension.

Recently, AI-based analytical methods, such as elastic net, weighted quantile sum (WQS) regression, and Bayesian kernel machine regression, have been suggested for estimating the effects of combined exposure to heavy metals [15–17]. These AI-based methods for estimating the effects of combined exposure to heavy metals involve the selection of the most important substances in mixture standardization, either through standardization based on ridge regression, least absolute shrinkage, and selection operator regression, or by constructing a weighted index and evaluating the assigned weights as the relative strength [16,17]. These methods are also used to compensate for potential nonlinearity in the estimation of combined exposure [16]. Although these methods enable the estimation of weights for the influence of individual heavy metals and the effects of combined exposure, if the mechanisms of interaction (positive, negative, and intermediate) within the mixture in the human body are not fully understood, there is much uncertainty in converting the impact of individual metals into weights. In this study, the authors estimated the effects of combined exposure to heavy metals by applying the statistical method of standardization used in the regression analysis. In the regression analysis, by converting variables with different units and distributions into standardized Z-scores using Ms and SDs, it is possible to estimate ORs by comparing the relative effects of different independent variables in terms of their standardized regression coefficients. Since non-standardized regression coefficients are affected by the scales (measurement units) of the independent variables, when the authors remove these effects through standardization, the authors can generalize the statistically distinct properties of different variables, such as time, units, and lethal doses. When designing learning models, such as the recent deep learning or machine learning models using AI, this statistical technique is very important in data preprocessing [18]. In addition, the authors did not identify any nonlinearity in the relationship between blood heavy metal levels and hypertension. Thus, the authors determined that the authors’ method using standardized Z-scores is statistically valid for estimating the effects of combined exposure to heavy metals.

Since the 1980s, many studies have reported the effects of heavy metals on hypertension [4]. In a recent cross-sectional study in Guangdong Province, China, a sample population with a hypertension prevalence of 36% was analyzed using the same covariates as those in the authors’ study. The ORs for hypertension associated with Pb and Cd exposure were reported to be 1.32 and 1.25, respectively, but these results were not statistically significant. In contrast, in a regression analysis that included waist circumference, fasting blood glucose, triglycerides, total cholesterol, and other heavy metals as covariates, significant differences were observed, with ORs of 1.38 for Pb and 1.42 for Cd [19]. Conversely, in another cross-sectional study on the relationship between blood heavy metal levels and hypertension in China, Cd was reported not to affect hypertension [20]. The relationship between heavy metals and resistant hypertension was analyzed using data from the US. In a multinomial logistic regression model, when analyzed by quartile, the ORs associated with Pb and Cd exposure were both 1.30. The WQS analysis showed significant combined effects for Pb, Cd, and Hg, with Pb showing the highest weight (0.64), followed by Cd (0.25) and Hg (0.11) [21]. In a study on CVD and urinary heavy metal levels, the prevalence of hypertension was 41.8%, and only Cd showed a significant OR for CVD (OR = 1.45), whereas Pb and Hg were not significant (OR = 0.80 and OR = 0.93, respectively). Meanwhile, in an additional analysis of the effects of heavy metals on CVD using the quantile g-computation, Cd, cobalt, and tungsten were significantly associated with CVD. At the same time, Pb was reported to reduce the risk of CVD [22]. In another study using data from the KNHANES in a sample population with a hypertension prevalence of 22.7%, the ORs for Pb and Cd were 1.91 and 2.36, respectively, indicating significant effects on hypertension [23]. Thus, reports on the relationship between heavy metals and hypertension differ depending on the sample population, ethnicity, and research method. However, among the heavy metals, the authors can surmise that Pb and Cd are closely related to hypertension and CVD. Nevertheless, there may be differences in sample populations depending on ethnicity, region, and sample design, and it will not be possible to accurately estimate ORs until the biomolecular mechanisms linking heavy metals and hypertension are fully understood. One limitation of this study is that regarding the relationships of individual heavy metals with hypertension prevalence, it was not possible to investigate synergistic or antagonistic effects. Nevertheless, as the authors evaluated the effects of not only exposure to single heavy metals but also combined exposure to several heavy metals, the authors were able to estimate the actual effects more precisely than those in previous studies that only considered individual heavy metals. These studies highlight the need to set tolerance levels for combined heavy metals and may provide clues for studying the health effects of unknown heavy metal interactions.

Heavy metals, including lead, mercury, and cadmium, are known to contaminate soil, water, and air and can accumulate in the human body through contact, ingestion, and inhalation. In particular, these metals are poorly excreted from the body and are associated with cardiovascular diseases [6]. Lead and Cd have different sources and contamination pathways, but are commonly exposed through paints, gasoline refineries, batteries, electronics, and tobacco [24,25]. Mercury, however, accounts for approx. two-thirds of the total emissions from the combustion of fossil fuels [26]. Globally, the Minamata Convention of the United Nations and the top 10 chemicals of major public health concern of the WHO emphasize regulations and cooperation on heavy metal emissions [27,28]. Heavy metal emissions from the body must be increased and neutralized using chelating agents, such as EDTA and antioxidant supplements [29,30]. Nonetheless, the chemical industry is still limited in its ability to regulate the hazards associated with human consumption, and exposure continues to expand with the development and expansion of new industrial technologies such as batteries, electronics, and semiconductors. Therefore, humanity needs to continue to monitor exposure to heavy metals from newly developed industrial technologies and to be able to estimate the effects of new mixtures of heavy metals on human health as industrial technologies evolve. Accurately estimating the effects of heavy metals is an important resource for planning preventive measures to reduce heavy metal exposure.

## CONCLUSIONS

The KNHANES introduced a rolling sample design in 2007, overcoming some limitations of cross-sectional analysis and integrating data across years to acquire a sufficient sample size. In addition, when weights are used in the process of data analysis, it is possible to correct for differences in household numbers and population counts at different sampling or survey time points and obtain a sample that is representative of the target population (i.e., the national population of South Korea), ensuring accuracy. In the study, the authors calculated weights by year, cohort, and survey items to accurately reflect the properties of the study population. The authors used regression analysis to estimate the effects of combined exposure to heavy metals. Thus, the authors estimated the effects of individual heavy metals and a combination of several heavy metals on hypertension and confirmed that Pb, Cd, and Hg are associated with hypertension. However, a limitation of this study is that Pb, Cd, and Hg were the only heavy metals included in the survey data, and the authors were unable to investigate the mechanisms or interactions responsible for the effects of heavy metals on hypertension. Further research is warranted to screen heavy metal-related risk factors for various diseases and to investigate the interactions involved in combined exposure to heavy metals.
